# Rapid identification of medically important mosquitoes by matrix-assisted laser desorption/ionization time-of-flight mass spectrometry

**DOI:** 10.1186/s13071-018-2854-0

**Published:** 2018-05-02

**Authors:** Abhishek Mewara, Megha Sharma, Taruna Kaura, Kamran Zaman, Rakesh Yadav, Rakesh Sehgal

**Affiliations:** 1 Department of Medical Parasitology, Postgraduate Institute of Medical Education and Research, Sector 12, Chandigarh, 160012 India; 2 Medical Microbiology, Postgraduate Institute of Medical Education and Research, Sector 12, Chandigarh, 160012 India

**Keywords:** MALDI-TOF MS, Mosquitoes, *Anopheles*, *Aedes*, *Culex*, *Armigerus*, ITS2 PCR, Rapid identification

## Abstract

**Background:**

Accurate and rapid identification of dipteran vectors is integral for entomological surveys and is a vital component of control programs for mosquito-borne diseases. Conventionally, morphological features are used for mosquito identification, which suffer from biological and geographical variations and lack of standardization. We used matrix-assisted laser desorption/ionization time-of-flight mass spectrometry (MALDI-TOF MS) for protein profiling of mosquito species from North India with the aim of creating a MALDI-TOF MS database and evaluating it.

**Methods:**

Mosquito larvae were collected from different rural and urban areas and reared to adult stages. The adult mosquitoes of four medically important genera, *Anopheles*, *Aedes*, *Culex* and *Armigerus*, were morphologically identified to the species level and confirmed by ITS2-specific PCR sequencing. The cephalothoraces of the adult specimens were subjected to MALDI-TOF analysis and the signature peak spectra were selected for creation of database, which was then evaluated to identify 60 blinded mosquito specimens.

**Results:**

Reproducible MALDI-TOF MS spectra spanning over 2–14 kDa m/z range were produced for nine mosquito species: *Anopheles* (*An*. *stephensi*, *An*. *culicifacies* and *An*. *annularis*); *Aedes* (*Ae*. *aegypti* and *Ae*. *albopictus*); *Culex* (*Cx*. *quinquefasciatus*, *Cx*. *vishnui* and *Cx*. *tritaenorhynchus*); and *Armigerus* (*Ar*. *subalbatus*). Genus- and species-specific peaks were identified to create the database and a score of > 1.8 was used to denote reliable identification. The average numbers of peaks obtained were 55–60 for *Anopheles*, 80–100 for *Aedes*, 30–60 for *Culex* and 45–50 peaks for *Armigeres* species. Of the 60 coded samples, 58 (96.67%) were correctly identified by MALDI-TOF MS with a score > 1.8, while there were two unreliable identifications (both *Cx*. *quinquefasciatus* with scores < 1.8).

**Conclusions:**

MALDI-TOF MS appears to be a pragmatic technique for accurate and rapid identification of mosquito species. The database needs to be expanded to include species from different geographical regions and also different life-cycle stages to fully harness the technique for entomological surveillance programs.

**Electronic supplementary material:**

The online version of this article (10.1186/s13071-018-2854-0) contains supplementary material, which is available to authorized users.

## Background

Mosquitoes are the most important dipteran vectors implicated in about 90% of all vector-borne diseases (VBD) infecting mankind [1]. Nearly one million people succumb to mosquito-transmitted diseases (MTD) every year worldwide [2]. While several endemic countries continue to struggle with the rampant malaria, filariasis and dengue, emergence and re-emergence of other VBDs in relatively naive geographical areas pose continuous threat to health services. A glaring example is that of chikungunya which has spread over the last 15 years with outbreaks reported across the globe [3]. Interestingly, a better adaptability of the virus to the switched over vector i.e. from *Aedes aegypti* to *Aedes albopictus*, contributed significantly to the massive spread of the disease [4]. Similarly, ongoing transmission of several other MTDs such as Zika virus disease, West Nile fever and yellow fever highlight the importance of studying the various factors associated with the related vectors [5, 6], to enable a better understanding of the transmission dynamics and institution of effective control measures. In the absence of a specific therapy or vaccines against most of the pathogens, vector control remains the mainstay for controlling the MTDs, where entomological surveys play a vital role.

The morphological identification of mosquitoes for delineation of genus and species based on specific taxonomical keys has long been the basis of entomological surveys. However, reliability of such identification remains questionable owing to loss of body structures during collection, transport and storage and more importantly due to presence of sibling-species and inter-species within the same species complex [7]. Molecular methods, mainly targeting the nuclear internal transcribed spacer 2 region (ITS2) and other gene targets for mosquito genera such as *Anopheles*, *Aedes* and *Culex* are the current gold standard for correct identification [8]. Although the molecular techniques are very sensitive and specific, DNA extraction from mosquitoes is a laborious process requiring technical expertise and dedicated personnel. Matrix-assisted laser desorption/ionization time-of-flight mass spectrometry (MALDI-TOF MS), with its unique ability of protein profiling, may provide an alternative to the morphological and molecular characterization of insects. After proving its worth in reliable identification of bacteria, fungi, giant viruses and Archea [9], the technique has been successfully employed for arthropods like *Drosophila* [10], aphids [11], *Culicoides* species [12], ticks [13], sand flies [14], tsetse flies [15], fleas [16] and also mosquitoes [8, 17–19]. Yssouf et al. [19] carried out MALDI-TOF MS based identification of 20 mosquito species prevalent in Senegal, created a database for them, and later on evaluated the robustness of the database by testing mosquitoes collected from Europe and added ten new species [20]. Few authors have also made use of cluster analysis to depict differences among the species of mosquitoes tested [8, 21]. MALDI-TOF MS based identification relies on the protein signatures of tested species and may show variations in response to environmental and other factors [14, 15, 22]. It is, therefore, pertinent to evaluate the MALDI-TOF MS protein profiles from different geographical regions. The current study thus aimed to harness this technique for protein profiling of the commonly encountered mosquito species in North India with the objective of creating a database and evaluating the robustness of the database by blinded-testing of mosquito specimens.

## Methods

### Mosquito larvae collection, maintenance and morphological identification

The aquatic breeding habitats of mosquitoes were surveyed from various rural, urban and slum areas of Chandigarh, a Union Territory in North India (30°73'33"N, 76°77'94"E), from March to October 2016. According to the size of a site, a representative number of dips were taken for collection using standard dipper (350 ml, minimum of 6 and maximum of 30 dips) from each type of water body from edges of sites, around vegetation, and shallow areas. Mosquito larvae of four genera, *Anopheles*, *Aedes*, *Culex* and *Armigerus*, were found to be prevalent. The anopheline breeding was found mostly in water collections and fields around cattle sheds, and edges of permanent rivers from August to October; *Culex* spp. were found mostly in parks, near vegetation, around cattle sheds and in ditches from May to October; *Armigeres* spp. were found more in dense vegetation with long persisting water logging from July to September; and *Aedes* spp. were found in water collections in parks, small streams, coolers, artificial containers and pots from March to September. The larvae were identified morphologically and separated in plastic trays on the basis of their genera. They were fed on a protein rich diet consisting of a mixture of finely powdered yeast extract and dog biscuits in the ratio of 3:2 and were reared up to the adult stage. All the adult mosquitoes were morphologically identified under the stereomicroscope (AO SteroStar, American Optical Corporation, New York, USA) using standard morphological keys [23], labeled and kept in separate Eppendorf tubes until further analysis.

### ITS2 polymerase chain reaction (PCR)

The DNA was extracted from legs of the adult mosquitoes using tissue DNA extraction kits as per the manufacturer’s instructions (Qiagen India Pvt. Ltd., New Delhi, India). The extracted DNA was amplified by PCR as previously described using specific primers targeting ITS2 region [24] (forward: 5'-TGT GAA CTG CAG GAC ACA T-3' and reverse: 5'-TAT GCT TAA ATT CAG GGG GT-3'). All the reagents used for PCR were obtained from Sigma-Aldrich (Sigma-Aldrich Chemicals Pvt. Ltd., Bangalore, India). The 50 μl reaction mixture contained 10 mM each of the forward and reverse primers, 100 mM each of the dNTPs, 2 U of *Taq* DNA polymerase and 2 μl of the extracted DNA. The amplification was performed in a thermocycler (Thermo Fisher Scientific, Vantaa, Finland) using the following parameters: initial denaturation at 94 °C for 5 min, 36 cycles each of denaturation at 94 °C for 1 min, annealing at 59 °C for 1 min, elongation at 72 °C for 1 min, followed by final extension at 72 °C for 5 min. PCR grade water was used as negative control in each reaction. The PCR products were analyzed by 1.2% agarose gel electrophoresis containing ethidium bromide and visualized in ultra-violet light in a gel documentation system (AlphaImager^TM^ EC, Protein-Simple, San Jose, CA, USA) to detect specific bands for different genera: 500 bp for *Anopheles* spp.; 300 bp for *Aedes* spp.; 450 bp for *Culex* spp.; and 420 bp for *Armigeres* spp.

The PCR products were sequenced using the Big-Dye Terminator sequencing kit in an ABI 3130 Genetic Analyzer automated sequencer (Applied Biosystems, Foster City, CA, USA) as per the manufacturer’s instructions. The sequencing primers were identical to the PCR primers.

### MALDI-TOF MS analysis

#### Sample preparation

The cephalothorax of the adult mosquitoes was selected for MALDI-TOF MS analysis. Briefly, each mosquito was examined under the stereomicroscope and its cephalothorax was carefully removed from the rest of the body (legs, wings, abdomen), and transferred to a 1.5 ml Eppendorf tube containing 20 μl of 50% formic acid. The cephalothorax was manually grinded with formic acid with the help of a fused tip. A solution containing 60% acetonitrile (ACN) and 0.3% tri-fluoroacetic acid (TFA) obtained from Sigma-Aldrich was prepared, and 7.5 μl of it was added to separate tubes. To this solution, 5 μl of the mosquito homogenate was added and mixed thoroughly. One microliter of this final solution was spotted onto the 96-well stainless steel plate, air dried, and overlaid with 1 μl of HCCA matrix (α-cyano-4-hydroxycinnamic acid) prepared by dissolving the matrix powder into a solution containing 50% ACN, 47.5% molecular-grade water and 2.5% TFA. Once the plate was completely dry, it was inserted into the port of Microflex MALDI-TOF MS (Bruker Daltonik GmbH, Bremen, Germany) to take the reading. Each specimen was spotted onto four different wells to check reproducibility. A blank tube with no mosquito parts in it underwent all the processing steps each time. *E*. *coli* ATCC 25922 was also spotted along with each plate to run as a control.

#### Parameters for MALDI-TOF MS analysis

Using the linear positive ion mode with a mass/charge (m/z) range of 2–20 kDa and the Flex Control software (Bruker Daltoniks), each well was subjected to 300 laser shots aimed at six different regions of the well with laser firing set at 50 shots/fire. All specimens belonging to the same species produced spectra that spanned over the 2–14 kDa m/z range with comparable peak patterns, intensity of peaks and the overall quality of spectra.

#### Spectra processing and database creation

Five morphologically and ITS2 PCR-confirmed female mosquitoes of each species were processed for creation of the database. The spectra were processed using the Flex Analysis software for peak smoothening and baseline subtraction. The spectra generated from four spots of the same specimen were compared using the ClinProTools v.2.2 software (Bruker Daltoniks) to check for reproducibility of the spectra. Out of these four spectra, the one having maximum number of peaks with > 50% intensity was selected. These selected spectra from all five representative specimens were used for creation of the database using Biotyper v.3.0. The same was done for all the nine species included in the study.

#### Identification of signature peaks for each species

The mass lists of all the specimens used for creation of the database, i.e. five female specimens per mosquito species, were exported to Microsoft Excel sheets for analysis. The peaks with a relative intensity of less than 5% were excluded, and those with a minimum signal-to-noise threshold of 4.0 and an aggregation of 800 ppm were selected. The optimal peaks thus obtained from the nine mosquito species were then compared to identify genus-specific and species-specific biomarker peaks among them.

#### Cluster analysis

A cluster analysis based on protein profiling was carried out to study the differences among the tested mosquito species with an anticipation that the closely related species will tend to cluster together, and away from the unrelated ones. This was done to check for the robustness of the database in identifying inter- and intra-species variations. One spectrum of each species was randomly chosen to create MSP (mean spectrum projection) for each of the nine mosquito species. Using Biotyper v.3.0, a dendrogram was constructed based on the principal components analysis (PCA).

#### Blinded-testing of coded mosquito specimens

Sixty fresh specimens collected from the same sites and confirmed by morphological and molecular identification by ITS2 PCR were coded and subjected to blinded-testing. Of the nine species, seven specimens of each species (except for *Ar*. *subalbatus* for which four specimens were available), were tested by MALDI-TOF MS. The protein extracted from their cephalothorax was spotted onto the 96-well stainless steel plate in triplicates and processed under Flex Control software. The acquired spectra were matched against the created database using Biotyper v.3.0. As described previously by Yssouf et al. [20] for European mosquito species, a score of > 1.8 was used to denote reliable genus and species identification, > 1.6 and < 1.79 to denote reliable genus identification, and a score of < 1.59 was considered unreliable.

## Results

### Mosquito larvae collection and identification

A total of 2000 mosquito larvae of the four genera were reared in the lab up to the adult stage for identification. Out of 1000 *Culex*, 600 *Aedes*, 200 *Armigeres* and 200 *Anopheles* larvae, 70, 59, 51, and 49% emerged into adults, respectively. A total of nine species were found: three species of *Anopheles* (*An*. *stephensi*, *An*. *culicifacies* and *An*. *annularis*); two species of *Aedes* (*Ae*. *aegypti*, and *Ae*. *albopictus*); three species of *Culex* (*Cx*. *quinquefasciatus*, *Cx*. *vishnui* and *Cx*. *tritaenorhynchus*); and one species of *Armigerus* (*Ar*. *subalbatus*). There was full concordance between morphological identification and ITS2 PCR sequencing for the identification of mosquito species.

### MALDI-TOF MS analysis

#### Analysis of signature peaks for different mosquitoes

**Fig. 1:**
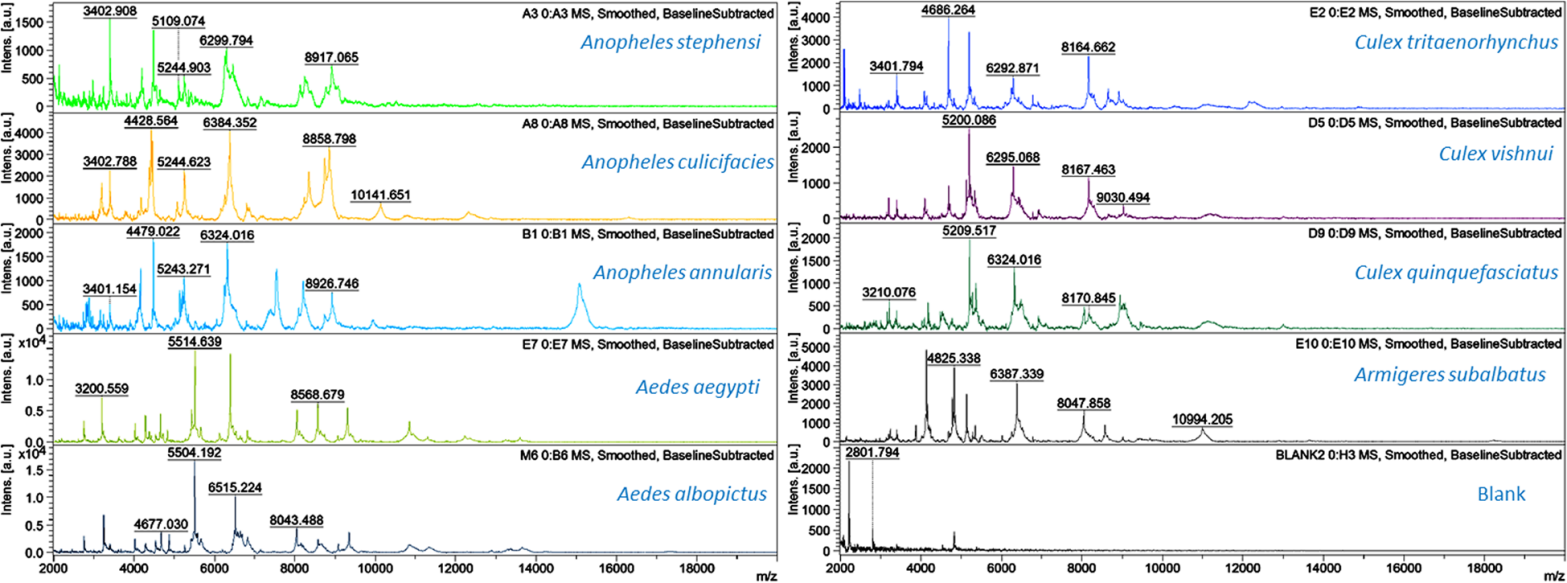
MALDI-TOF MS spectra obtained from the cephalothorax part of nine mosquito species

The average numbers of peaks produced by five female specimens of the different mosquito species were 55–60 peaks for *Anopheles*, 80–100 for *Aedes*, 30–60 for *Culex* and 45–50 for *Armigeres* species (Fig. 1, Additional file 1: Figures S1-S9). The optimal peaks selected from among these are represented in Table 1. A few genus-specific signature peaks present in all species of the same genus were identified. For example, peak (m/z) 5243 was present in all anopheline species, 9310 in both aedine species, and 8167 in all culicine species. Among the three species of *Anopheles*, peaks 6298 and 8915 were present only in *An*. *stephensi*; 3192, 4428, 6383, 8858 and 10144 only in *An*. *culicifacies*; and 8924 only in *An*. *annularis*; while peak 4480 was present in both *An*. *stephensi* and *An*. *annularis*, but not in *An*. *culicifacies*. Among the two species of *Aedes*, wide variation among signature peaks was present. Among the three *Culex* species, peak 9028 was specific for *Cx*. *vishnui*, 4686 for *Cx*. *tritaenorhynchus*, and 3210 and 5210 for *Cx*. *quinquefasciatus*, while peaks 5198 and 6293 were present in both *Cx*. *tritaenorhynchus* and *Cx*. *vishnui*, but not in *Cx*. *quinquefasciatus*. The biomass peaks 4825, 5138, 6387, 8578 and 10995 were present only in *Ar*. *subalbatus*.

**Table 1:** Representative biomarker peaks for nine mosquito species as obtained by MALDI-TOF MS analysis

**Biomass**	***An. st.***	***An. cu.***	***An. an.***	***Ae. ae.***	***Ae. al.***	***Cx. tr.***	***Cx. vi.***	***Cx. qu.***	***Ar. su.***
3192		×							
3200				×					
3210								×	
3402	×	×	×			×			
4428		×							
4480	×		×						
4675					×				
4686						×			
4825									×
4831				×					
5138									×
5198						×	×		
5210								×	
5243	×	×	×						
5505					×				
5514				×					
6293						×	×		
6298	×								
6324			×					×	
6383		×							
6387									×
6399				×					
6515					×				
8042					×				×
8056				×					
8167						×	×	×	
8565				×					
8578									×
8858		×							
8915	×								
8924			×						
9028							×		
9310				×	×				
10144		×							
10995									×

*Abbreviations*: *Ae. ae. Aedes aegypti*, *Ae. al. Aedes albopictus*, *An. an. Anopheles annularis*, *An. cu. Anopheles culicifacies*, *An. st. Anopheles stephensi*, *Ar. su. Armigeres subalbatus*, *Cu. qu. Culex quinquefasciatus*, *Cu. tr. Culex tritaenorhynchus*, *Cu. vi. Culex vishnui*

#### Cluster analysis

**Fig. 2:**
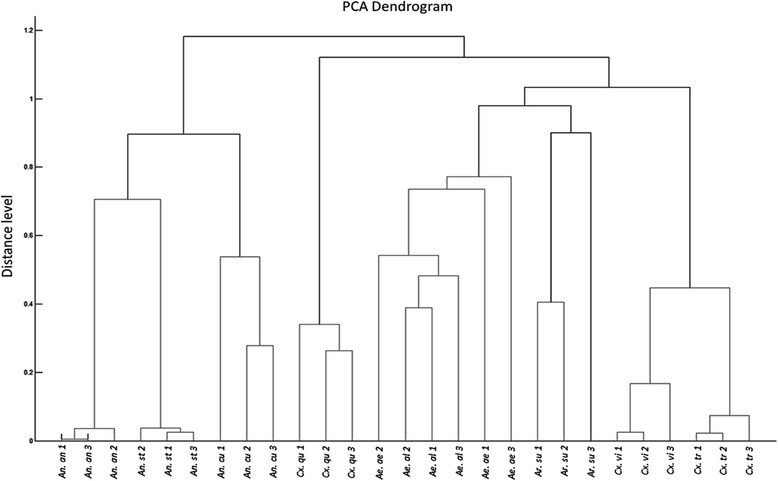
Dendrogram constructed by Biotyper for nine mosquito species. Numbers 1, 2 and 3 denote labels of three specimens of each species of mosquito. The cephalothorax part of the mosquitoes was taken for analysis. *Abbreviations*: *Ae*. *ae*, *Aedes aegypti*; *Ae*. *al*, *Aedes albopictus*; *An*. *an*, *Anopheles annularis*; *An*. *cu*, *Anopheles culicifacies*; *An*. *st*, *Anopheles stephensi*; *Ar*. *su*, *Armigeres subalbatus*; *Cu*. *qu*, *Culex quinquefasciatus*; *Cu*. *tr*, *Culex tritaenorhynchus*; *Cu*. *vi*, *Culex vishnui*

The three species of *Anopheles* (*An*. *stephensi*, *An*. *annularis* and *An*. *culicifacies*) clustered together. The two *Culex* species (*Cx*. *vishnui* and *Cx*. *tritaenorhynchus)* clustered together while *Cx*. *quinquefasciatus* stood out from the other species. *Armigeres* clustered closer to *Aedes* than any other species (Fig. 2).

#### Analysis of blinded-testing of coded mosquitoes

Of the 60 coded mosquito specimens, 58 (96.67%) were correctly identified with the database with a score of > 1.8, while 2 specimens (both confirmed as *Cx*. *quinquefasciatus* by ITS2 PCR) were misidentified as *Cx*. *vishnui*. Both these specimens produced an unreliable identification with a score of < 1.6, even on repeated testing on the same wells in addition to different wells with the same homogenates. The blank always produced a score of < 1 and the *E*. *coli* ATCC 25922 gave a reliable score of > 2 every time when matched against the in-built database of the Biotyper.

## Discussion

In this study, we collected four genera of medically important mosquitoes from North India and used them for creating MALDI-TOF MS protein profiles for rapid and accurate identification. While for *Ae*. *aegypti*, *Ae*. *albopictus*, *An*. *stephensi* and *Cx*. *quinquefasciatus*, a database has been recently created [8, 19], the other five species, i.e. *An*. *annularis*, *An*. *culicifacies*, *Cx*. *vishnui*, *Cx*. *tritaenorhynchus* and *Ar*. *subalbatus* have not yet been evaluated by MALDI-TOF MS. The anophelines are known vectors of malaria, aedine species spread dengue and chikungunya, culicines spread filariasis (*Cx*. *quinquefasciatus*), Japanese encephalitis and West Nile fever (*Cx*. *vishnui*, *Cx*. *tritaenorhynchus*), and *Ar*. *subalbatus* is implicated in filariasis and Japanese encephalitis. These MTDs pose significant public health concerns worldwide as well as in India. The quick and reliable identification of these mosquito species is essential to implement effective vector control measures, in addition to having important epidemiological significance [25].

The identification using MALDI-TOF MS is based on signature proteins of the organism and different life-cycle stages of mosquitoes will generate different mass spectra [26, 27]. We collected larvae from the field, reared them to adult stage, and used the cephalothorax for protein extraction. Muller et al. [8] also used cephalothorax for identifying *An*. *gambiae* mosquitoes; however, different body structures like legs of mosquitoes [19], or even different life-cycle stages like larvae [28] and eggs [18] of mosquitoes have been previously used, while the abdomen of the adult mosquito is not preferred as it may contain the residual meal of the insect which may interfere with the protein peaks [11, 21, 29]. Yssouf et al. [19] have reported good results by using mosquito legs for protein extraction; however, we preferred to use the cephalothorax as it adequately yields the minimum concentration of 0.2 mg/ml of raw protein required for generation of optimal spectrum by MALDI-TOF MS [28]. The legs of the mosquito are less likely to be contaminated with extraneous proteins; however, some of the legs can easily be lost during collection, transportation or storage of mosquitoes, thereby decreasing the protein content. Furthermore, in the present study, we used the legs of the mosquitoes for molecular identification and the cephalothorax of the same mosquitoes for MALDI-TOF analysis. The turn-around time was less than 30 minutes per specimen and the running cost was less than USD 0.05 per specimen, excluding the cost of the machine.

The spectra generated by the nine mosquito species were not only visually distinct but also had specific signature peaks. Of the species we tested, Steinmann et al. [28] have documented the peaks obtained for larvae of *Ae*. *aegypti*; while some of their peaks coincided with *Ae*. *aegypti* in our study, the difference in other peaks was anticipated due to the different life stage of mosquito analyzed (larva *vs* adult). Yssouf et al. [19] have obtained peaks from the legs of *Ae*. *aegypti* and *Cx*. *quinquefasciatus* and their peaks vary greatly from those in our study. This could be attributed to the difference in the body part used for analysis in the two studies, as also described earlier by Karger et al. [22] for ticks. Spectral differences have also been previously noted when different body parts (cephalothorax *vs* legs) of flies were tested and may be attributed to difference in the protein content [15, 21]. The differences could also be due to intra-specific genetic diversity among specimens collected from different environments and geographical locations, as reported earlier in anopheline species [30], *Ae*. *aegypti* [31], *Ae*. *albopictus* [32] and *Cx*. *quinquefasciatus* [33], based on molecular gene targets, and it is likely that their protein profiles may also exhibit variations. Further studies investigating the role of environmental and host factors on genetic and phenotypic variations among members of the same species can contribute to our knowledge of such diversity.

The dendrogram analysis of the nine species clustered them on the basis of their mass spectra similarities. As expected, three of the anophelines (*An*. *stephensi*, *An*. *culicifacies* and *An*. *annularis*) clustered together. It was observed that the similarity between *An*. *stephensi* and *An*. *annularis* was more than that with *An*. *culicifacies*. This corroborates with the reported genomic linkage in which *An*. *stephensi* and *An*. *annularis* cluster together on the basis of ITS2 spacer and *cox*2 gene while *An*. *culicifacies* only shows some linkage with *An*. *annularis* on the basis of the *NOS* gene [30]. Both aedine species, as expected on the basis of their genomic similarity [34], clustered closely together. Among the culicine species, *Cx*. *quinquefasciatus* aligned away from *Cx*. *vishnui* and *Cx*. *tritaenorhynchus*; while it was expected owing to a unique set of peaks produced only by *Cx*. *quinquefasciatus*, this finding could also be plausibly explained on the basis of the genetic diversity of *Cx*. *quinquefasciatus* which forms a separate clade from the rest of the *Culex* species [35]. The last species, *Ar*. *subalbatus*, a relatively less-studied species, clustered along with aedine species instead of forming a separate subgroup. Constituting nearly 7% of all mosquito species in central India, this species blooms during monsoons with sewage and dirty water as its major habitat [36]. Although vast geographical variation has been reported [37], the low heterozygosity between *Armigeres* and *Aedes* [38] may explain its clustering along with the aedine species.

After creating the database, blinded testing of 60 specimens was done to analyze the reliability of identification (using 1.8 as the cut-off score). Yssouf et al. [19] have used a cut-off of 1.8 for mosquitoes, and 1.7 for ticks [13], although no general consensus exists regarding the optimal cut-off for vector identification by MALDI-TOF MS as of now. With a cut-off of 1.8, 96.67% of the coded specimens were correctly identified by our database. Two *C*. *quinquefasciatus* scored poorly even on repeated testing, the reasons of which need further exploration. Similar observation has been made earlier where unreliable identification (score < 1.8) of three mosquito species (*Culiseta longiareolata*, *Coquillettidia richiardii* and *Ae*. *caspius*) was attributed to lower spectral quality [20]. More prospective studies will help to validate the database and to define optimal cut-offs for reliable vector identification.

MALDI-TOF MS is now being heavily relied upon for the identification of bacteria and other microorganisms; however, its reliable use for identification of vectors encompasses many challenges. First, to enable construction of a reliable database as that for other pathogens, it is important to take into consideration several parameters affecting protein profiles in a vector: life-cycle stage, body part, gender differences, genetic diversity, environmental and geographical variations, extraction protocol, etc. Complex as it appears, this might be the reason why the MALDI-TOF machines do not have in-built databases for vectors until now. The Switzerland-based firm, Mabritec AG, has created a database including 70 arthropod species [7]; however, it needs to be meticulously validated on species collected from across the globe. Secondly, as the identification depends upon protein profiling, it is deemed necessary that the integrity of protein is maintained during collection, transportation, storage and processing of the specimen. Finally, with the known and ongoing genetic diversity occurring within sibling-species of mosquitoes, a regular update of the databases may be necessary. These limitations should be overcome with time as more data is accumulated in this field.

## Conclusions

MALDI-TOF MS appears to be a pragmatic, rapid, accurate and cost-effective tool for the identification of mosquitoes which may circumvent the redundancies of morphological variations as well as the complexities of DNA-based identification tools. However, more information is required before standardized protocols can be created for vectors. Its utility can be expanded to include important arenas like characterization of insecticide resistance in vectors, detection of carriage of pathogens, and also study of dipteran vectors other than mosquitoes. Thus, MALDI-TOF MS has great potential for the study of vectors and it is worthwhile to accrue more information on its use to enhance the clarity of the scope of its applicability in the control of vector-borne diseases.

## Additional file

Additional file 1: **Figure S1.** MALDI-TOF MS spectra of three representative specimens of *An. stephensi*. **Figure S2.** MALDI-TOF MS spectra of three representative specimens of *An. culicifacies*. **Figure S3.** MALDI-TOF MS spectra of three representative specimens of *An. annularis*. **Figure S4.** MALDI-TOF MS spectra of three representative specimens of *Ae. aegypti*. **Figure S5.** MALDI-TOF MS spectra of three representative specimens of *Ae. albopictus*. **Figure S6.** MALDI-TOF MS spectra of three representative specimens of *Cx. tritaenorhynchus*. **Figure S7.** MALDI-TOF MS spectra of three representative specimens of *Cx. vishnui*. **Figure S8.** MALDI-TOF MS spectra of three representative specimens of *Cx. quinquefasciatus*. **Figure S9.** MALDI-TOF MS spectra of three representative specimens of *Ar. subalbatus*. (PDF 2050 kb)

## Abbreviations

ACN: Acetonitrile
HCCA: α-cyano-4-hydroxycinnamic acid
ITS: Internal transcribed spacer
MALDI-TOF MS: Matrix-assisted laser desorption/ionization time-of-flight mass spectrometry
MTD: Mosquito-transmitted diseases
PCA: Principal components analysis
PCR: Polymerase chain reaction
TFA: Tri-fluoroacetic acid
VBD: Vector-borne diseases
