# Supplementary material for: Rapid identification of medically important mosquitoes by matrix-assisted laser desorption/ionization time-of-flight mass spectrometry
Source: Parasit Vectors. 2018 May 2;11:281. doi: 10.1186/s13071-018-2854-0 (PMC5932809; doi:10.1186/s13071-018-2854-0)
Supplement: Supplementary file 1 — Figure S1. MALDI-TOF MS spectra of three representative specimens of An. stephensi. Figure S2. MALDI-TOF MS spectra of three representative specimens of An. culicifacies. Figure S3. MALDI-TOF MS spectra of three representative specimens of An. annularis. Figure S4. MALDI-TOF MS spectra of three representative specimens of Ae. aegypti. Figure S5. MALDI-TOF MS spectra of three representative specimens of Ae. albopictus. Figure S6. MALDI-TOF MS spectra of three representative specimens of Cx. tritaenorhynchus. Figure S7. MALDI-TOF MS spectra of three representative specimens of Cx. vishnui. Figure S8. MALDI-TOF MS spectra of three representative specimens of Cx. quinquefasciatus. Figure S9. MALDI-TOF MS spectra of three representative specimens of Ar. subalbatus. (PDF 2050 kb) [file 13071_2018_2854_MOESM1_ESM.pdf]

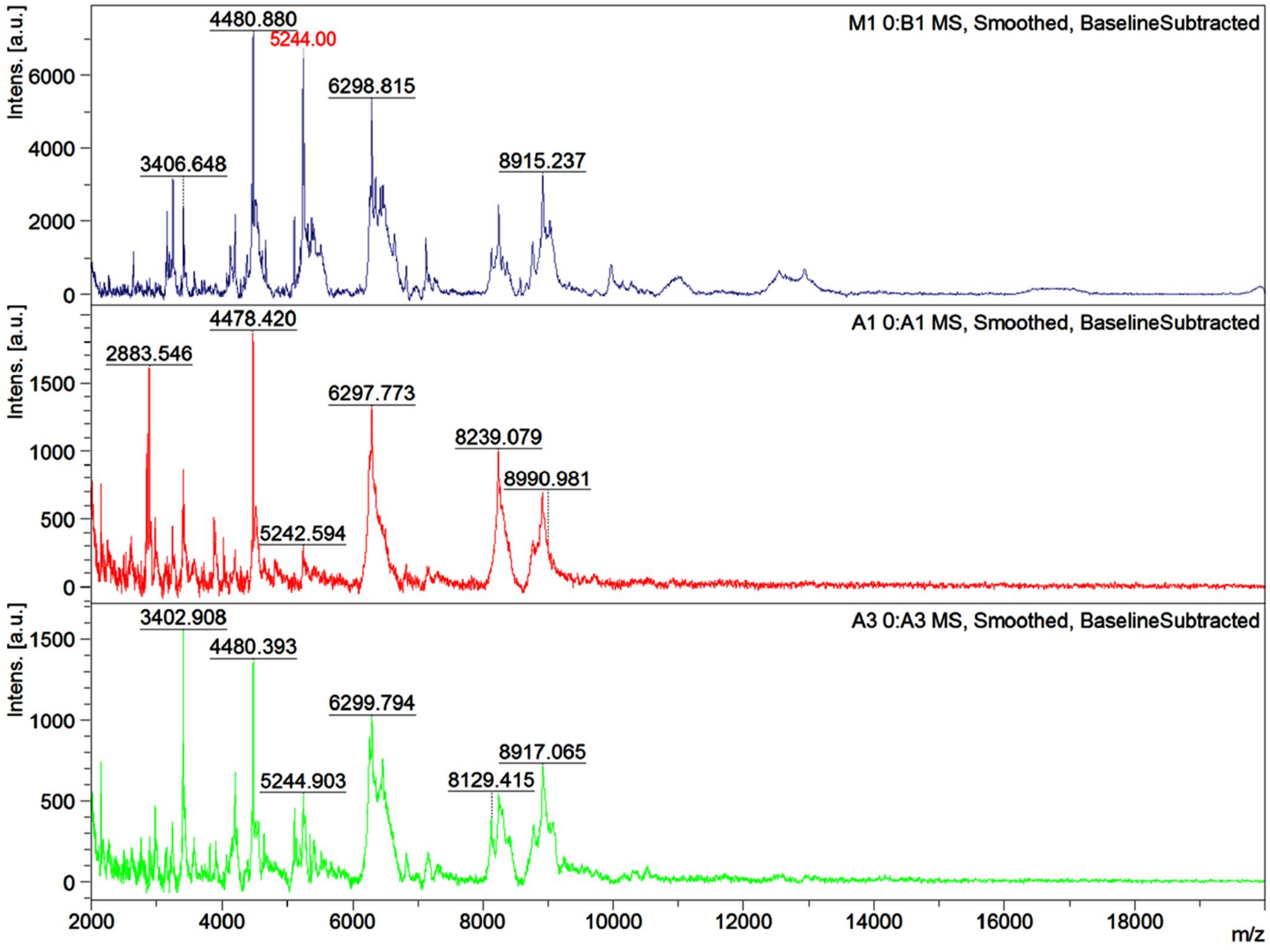

Figure S1. MALDI-TOF MS spectra of three representative specimens of *An. stephensi*.

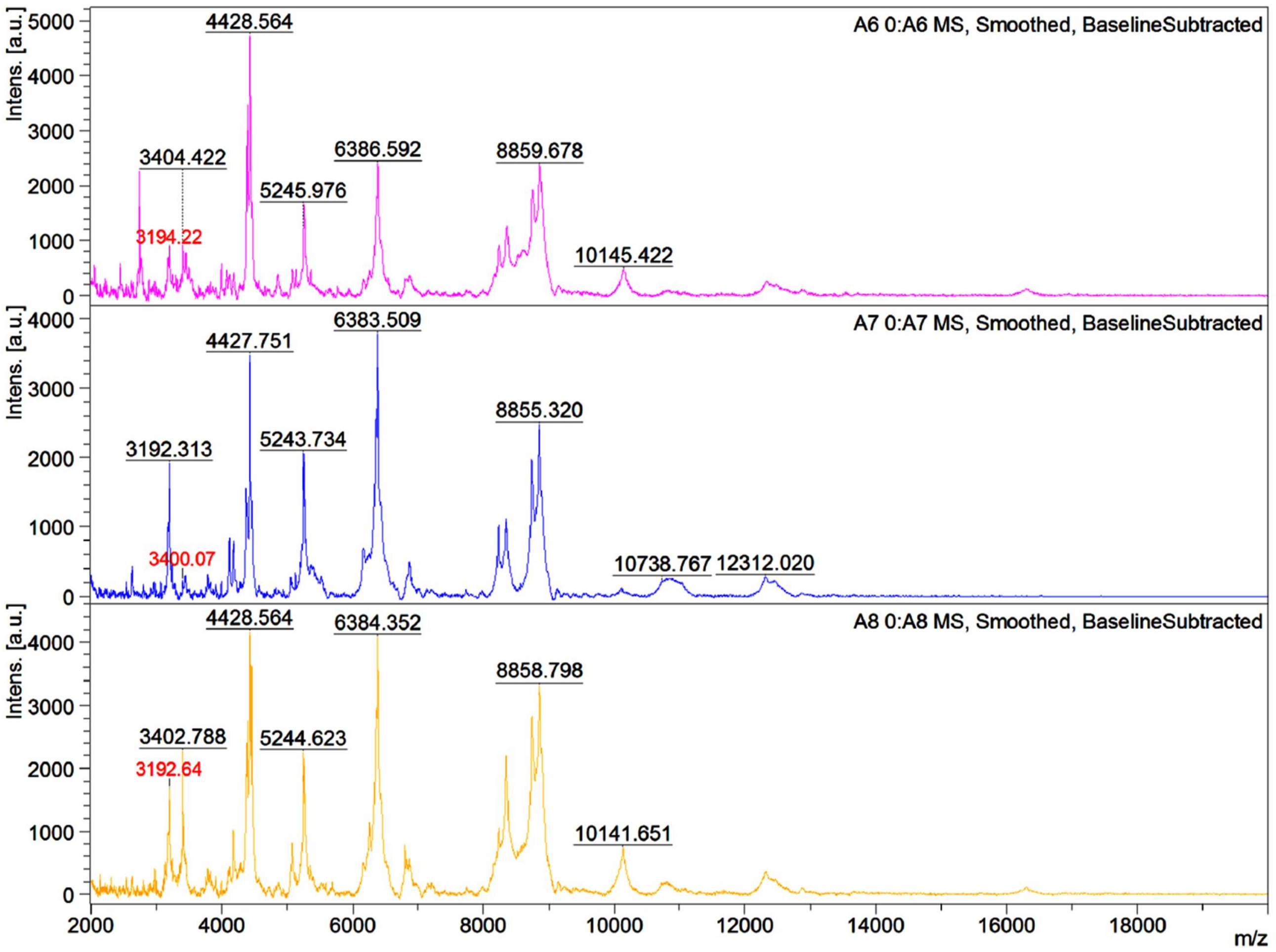

Figure S2. MALDI-TOF MS spectra of three representative specimens of *An. culicifacies*.

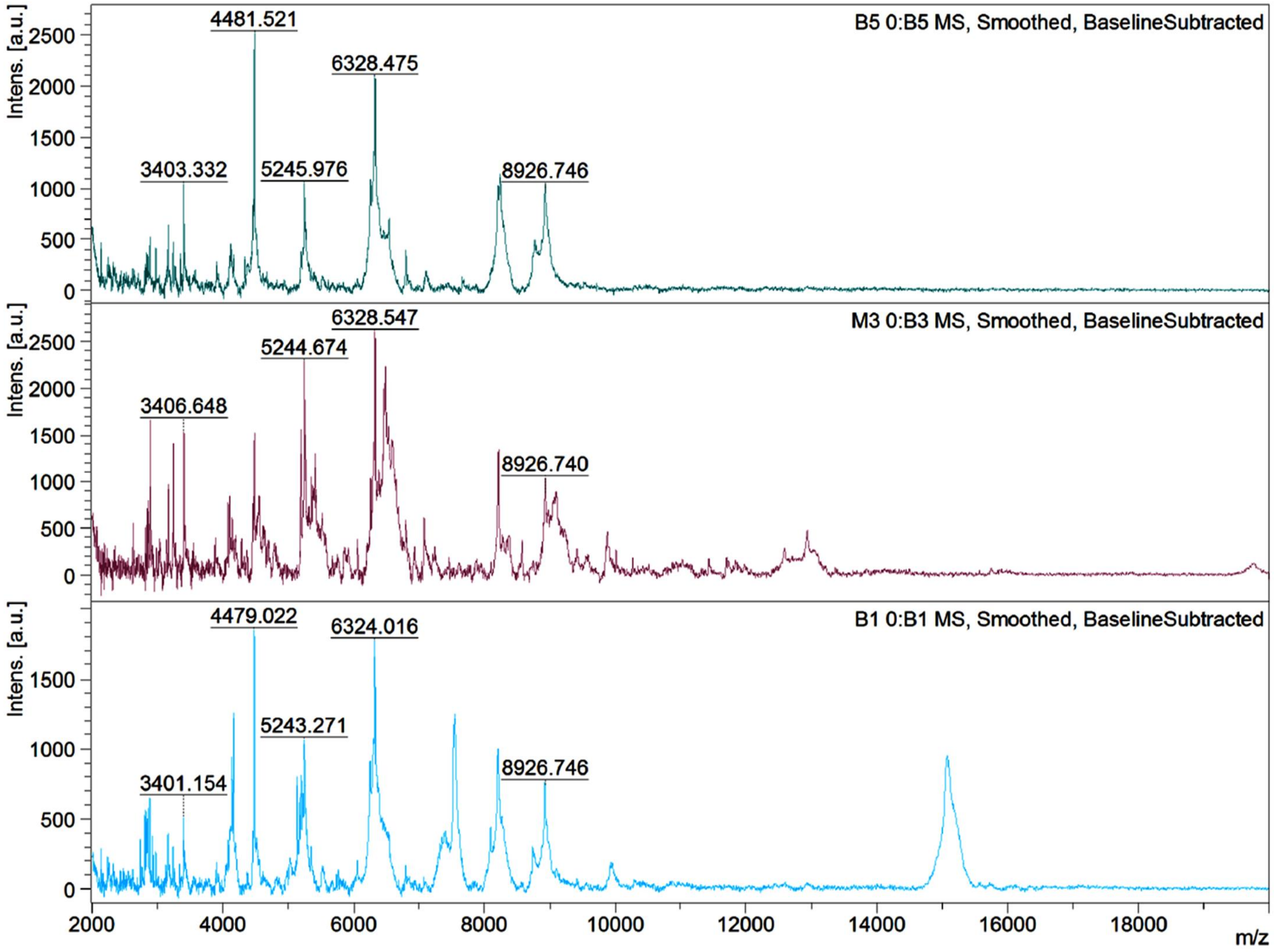

Figure S3. MALDI-TOF MS spectra of three representative specimens of *An. annularis*.

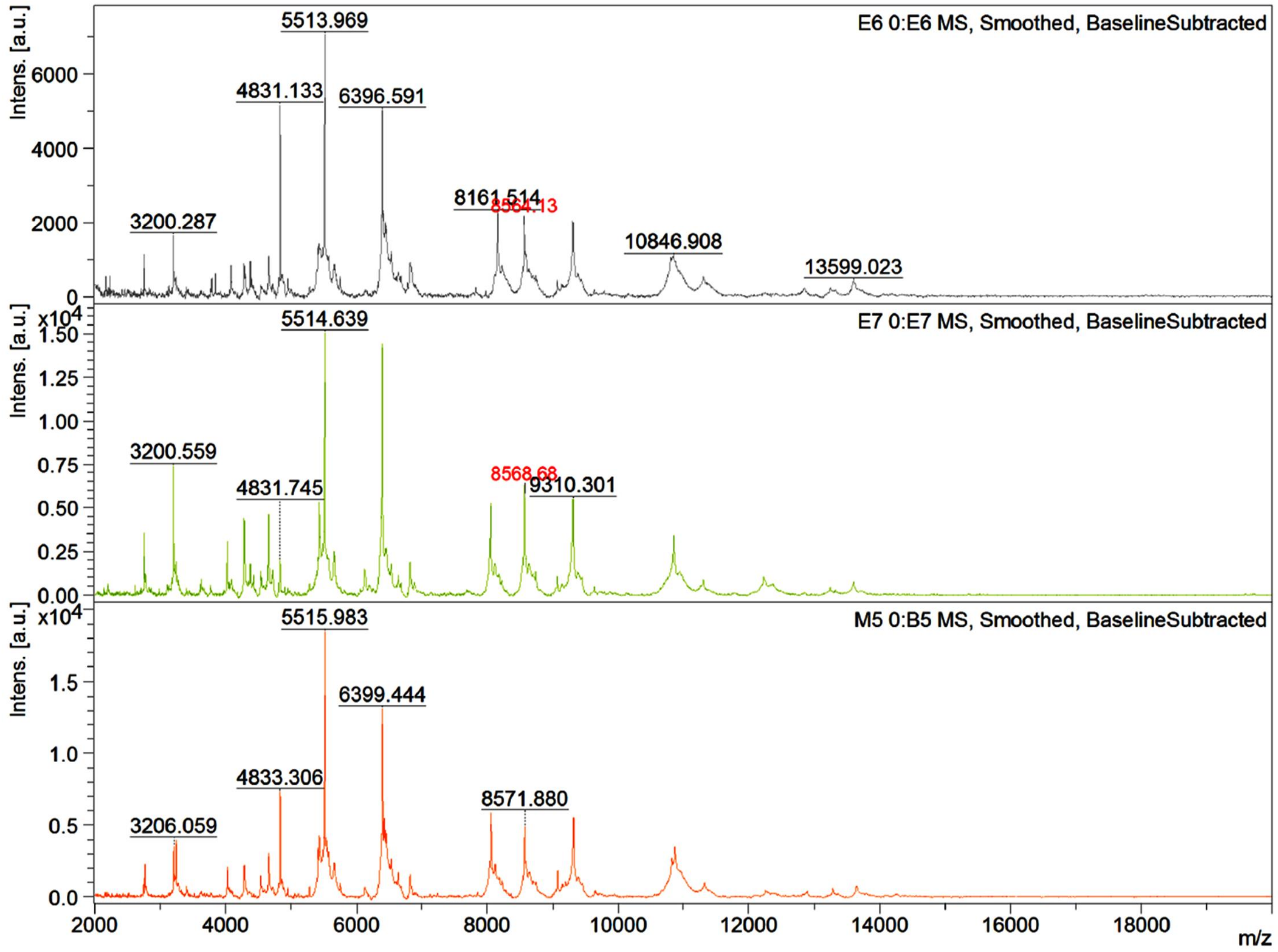

Figure S4. MALDI-TOF MS spectra of three representative specimens of *Ae. aegypti*.

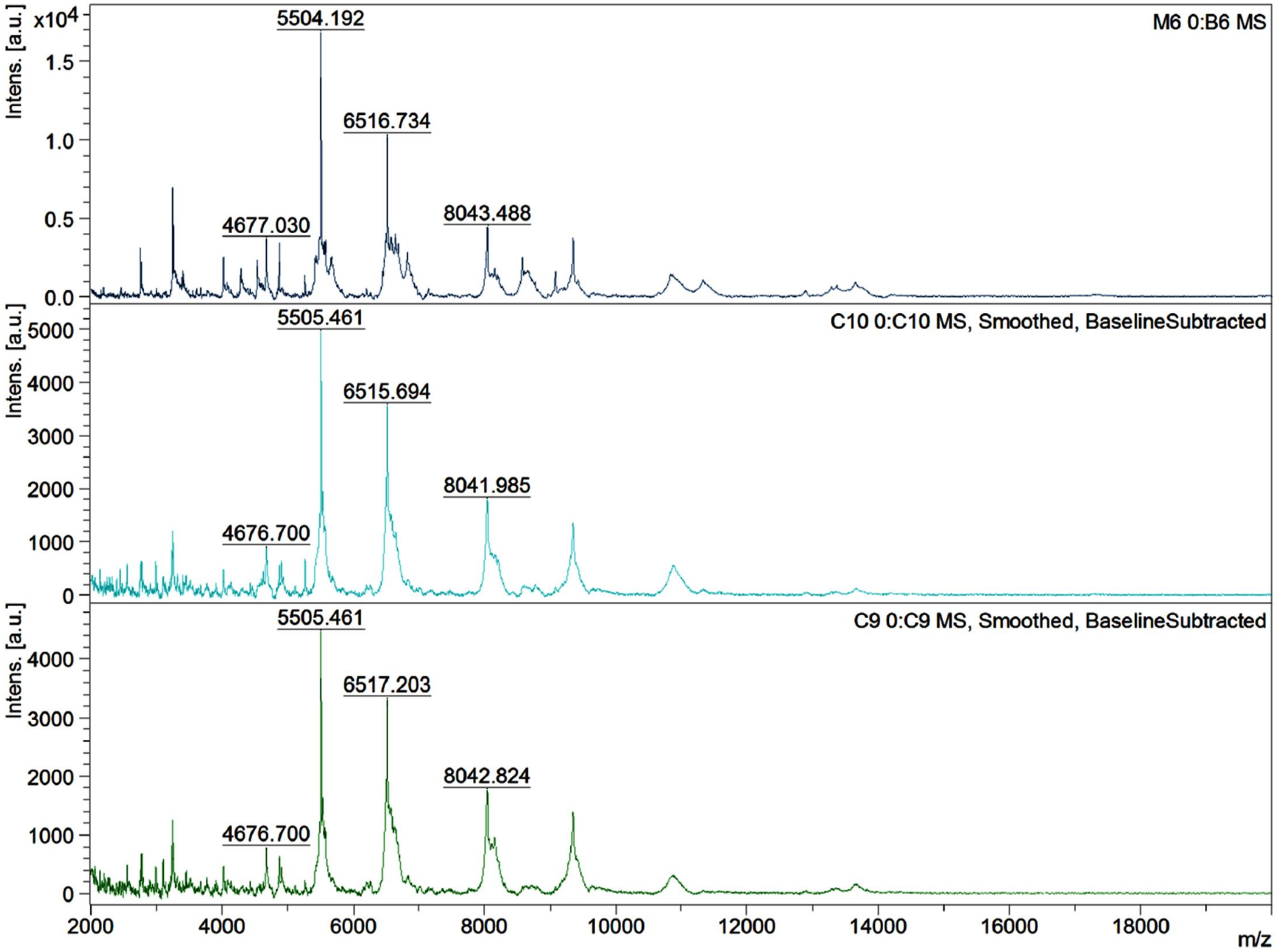

Figure S5. MALDI-TOF MS spectra of three representative specimens of *Ae. albopictus*.

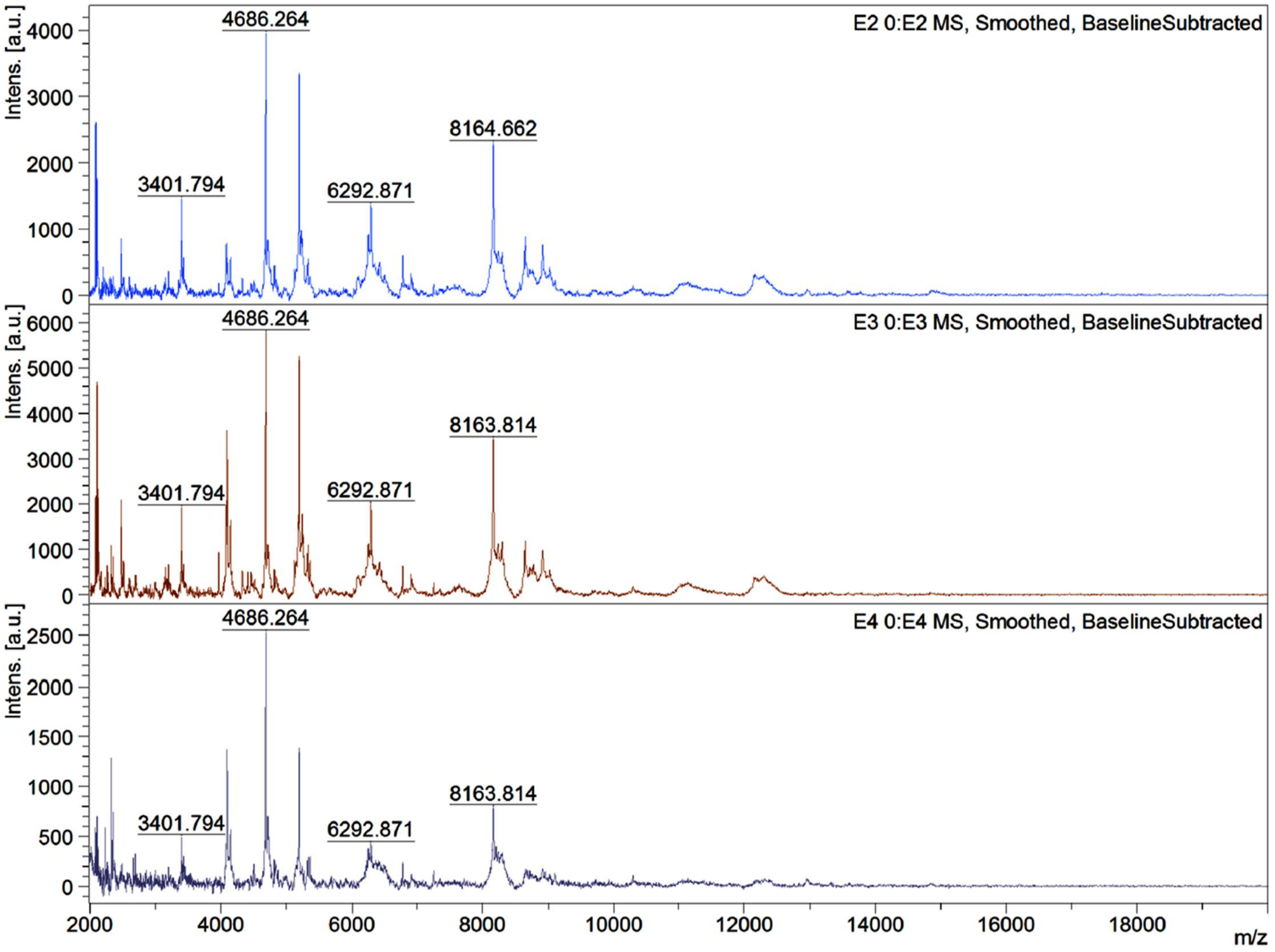

Figure S6. MALDI-TOF MS spectra of three representative specimens of *Cx. tritaenorhynchus*.

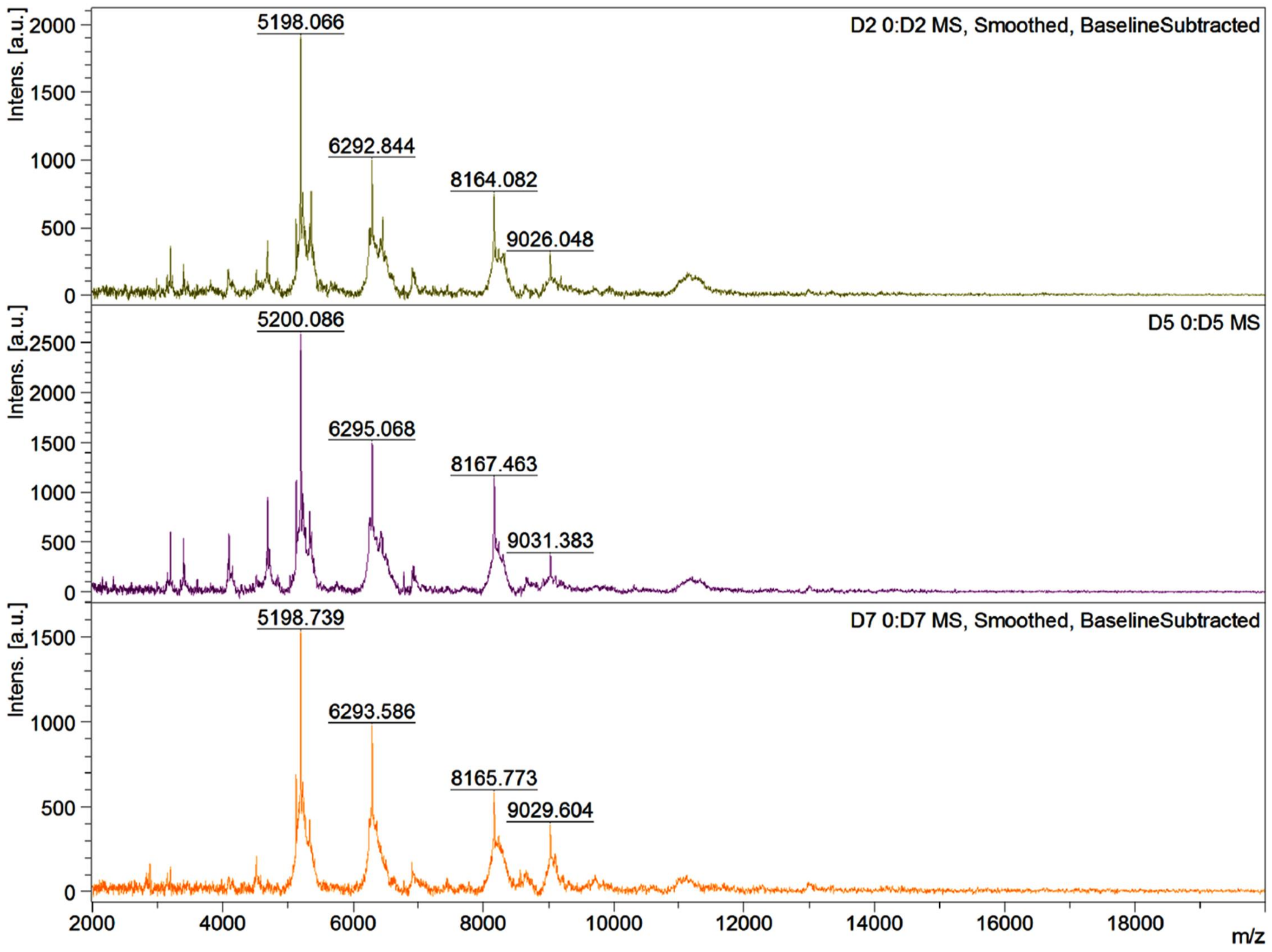

Figure S7. MALDI-TOF MS spectra of three representative specimens of *Cx. vishnui*.

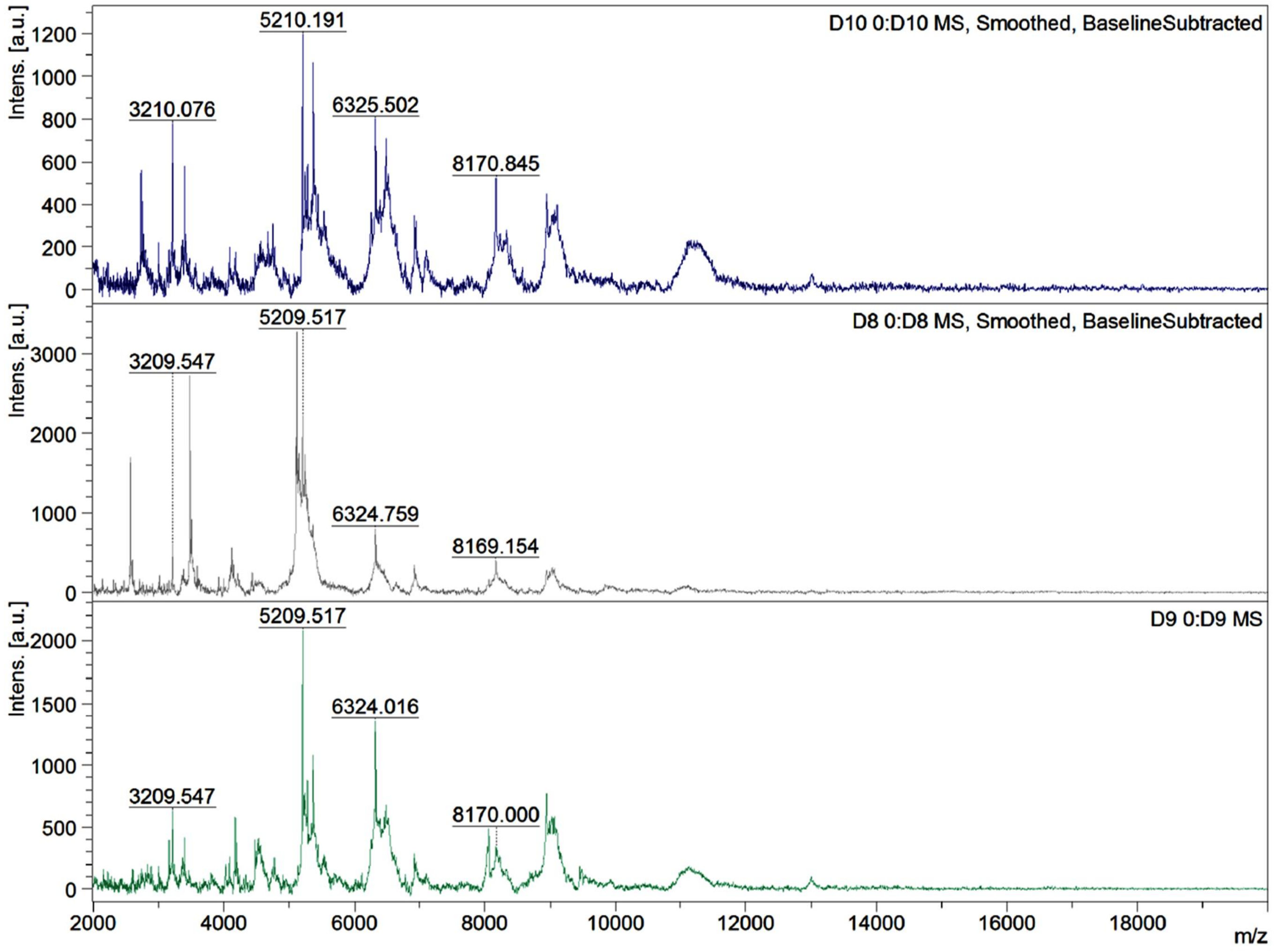

Figure S8. MALDI-TOF MS spectra of three representative specimens of *Cx. quinquefasciatus*.

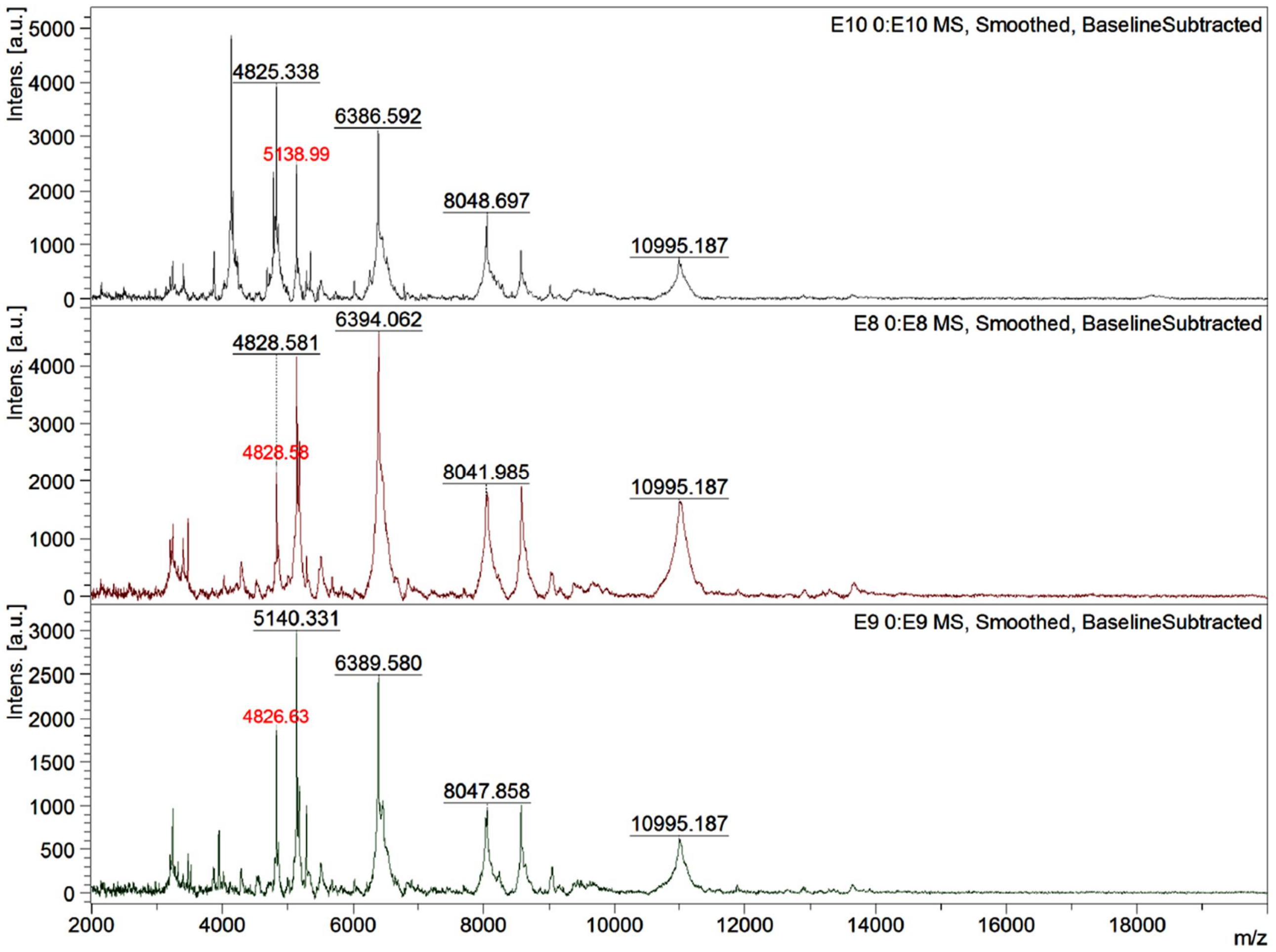

Figure S9. MALDI-TOF MS spectra of three representative specimens of *Ar. subalbatus*.
